# Induction of complementary immunogenic necroptosis and apoptosis cell death pathways inhibits cancer metastasis and relapse

**DOI:** 10.21203/rs.3.rs-3992212/v1

**Published:** 2024-03-12

**Authors:** Christopher Egbulefu, Kvar Black, Xinming Su, Partha Karmakar, LeMoyne Habimana-Griffin, Gail Sudlow, Julie Prior, Ezeugo Chukwu, Alex Zheleznyak, Baogang Xu, Yalin Xu, Alison Esser, Matthew Mixdorf, Evan Moss, Brad Manion, Nathan Reed, Matthew Gubin, Chieh-Yu Lin, Robert Schreiber, Katherine Weilbaecher, Samuel Achilefu

**Affiliations:** 1 Department of Radiology, Washington University in St. Louis, MO 63110, USA.; 2 Department of Medicine, Washington University in St. Louis, MO 63110, USA.; 3 Department of Pathology and Immunology, Washington University in St. Louis, MO 63110, USA.; 4 Department of Biomedical Engineering, Washington University in St. Louis, MO 63110, USA.; 5 Department of Biomedical Engineering, The University of Texas Southwestern Medical Center, Dallas, TX 75235-9397, USA

## Abstract

Interactions of light-sensitive drugs and materials with Cerenkov radiation-emitting radiopharmaceuticals generate cytotoxic reactive oxygen species (ROS) to inhibit localized and disseminated cancer progression, but the cell death mechanisms underlying this radionuclide stimulated dynamic therapy (RaST) remain elusive. Using ROS-regenerative nanophotosensitizers coated with a tumor-targeting transferrin-titanocene complex (TiO_2_-TC-Tf) and radiolabeled 2-fluorodeoxyglucose (^18^FDG), we found that adherent dying cells maintained metabolic activity with increased membrane permeabilization. Mechanistic assessment of these cells revealed that RaST activated the expression of RIPK-1 and RIPK-3, which mediate necroptosis cell death. Subsequent recruitment of the nuclear factors kappa B and the executioner mixed lineage kinase domain-like pseudo kinase (MLKL) triggered plasma membrane permeabilization and pore formation, respectively, followed by the release of cytokines and immunogenic damage-associated molecular patterns (DAMPs). In immune-deficient breast cancer models with adequate stroma and growth factors that recapitulate the human tumor microenvironment, RaST failed to inhibit tumor progression and the ensuing lung metastasis. A similar aggressive tumor model in immunocompetent mice responded to RaST, achieving a remarkable partial response (PR) and complete response (CR) with no evidence of lung metastasis, suggesting active immune system engagement. RaST recruited antitumor CD11b^+^, CD11c^+^, and CD8b^+^ effector immune cells after initiating dual immunogenic apoptosis and necroptosis cell death pathways in responding tumors in vivo. Over time, cancer cells upregulated the expression of negative immune regulating cytokine (TGF-β) and soluble immune checkpoints (sICP) to challenge RaST effect in the CR mice. Using a signal-amplifying cancer-imaging agent, LS301, we identified latent minimal residual disseminated tumors in the lymph nodes (LNs) of the CR group. Despite increased protumor immunogens in the CR mice, RaST prevented cancer relapse and metastasis through dynamic redistribution of ROS-regenerative TiO_2_ from bones at the early treatment stage to the spleen and LNs, maintaining active immunity against cancer progression and migration. This study reveals the immune-mechanistic underpinnings of RaST-mediated antitumor immune response and highlights immunogenic reprogramming of tumors in response to RaST. Overcoming apoptosis resistance through complementary necroptosis activation paves the way for strategic drug combinations to improve cancer treatment.

## Graphical Abstract

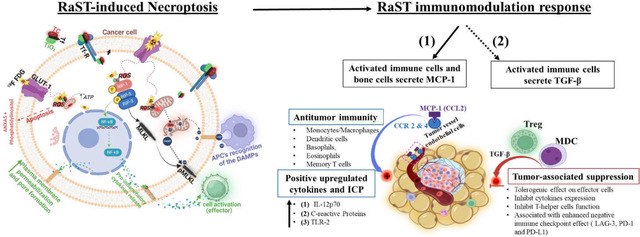

## INTRODUCTION

Aggressive cancer cells exhibit multiple intrinsic and extrinsic mechanisms to evade the cytotoxic effects of drugs, leading to increased morbidity and mortality.^1–3^ This rapid adaptation to treatment and the extensive heterogeneity of cancer cells underscores the challenges in selecting appropriate drug combinations to eradicate them. While some primary tumors respond to chemotherapeutics, the prognosis for disseminated diseases such as metastatic breast cancer remains bleak.^4^ In breast cancer, the highly immunosuppressive tumor microenvironment undermines the benefits of immunotherapy demonstrated in other cancer types.^5,6^ Combining standard treatment with an alternative method to induce an antitumor immune response could improve treatment outcomes, especially for metastatic disease. A viable approach is to use photophysical interventions in cancer treatment.

Traditional photodynamic therapy (PDT), which uses an external light source to excite photosensitizers in tumors and generate reactive oxygen species (ROS), can induce the expression of tumor antigens or cell death immunogens for adjuvant antitumor effect.^7–11^ Due to the limited light penetration in tissue, the application is confined to lesions that are locally accessible to direct light stimulation. Previously, we and others reported a phenomenon where radionuclides capable of emitting Cerenkov radiation can interact with photosensitizers to generate cytotoxic ROS for cancer treatment.^12,13^ This radionuclide-stimulated dynamic therapy (RaST) exerts its toxic effect when both the photosensitizer and the radiopharmaceutical converge in tumors.^12,13^ Using 2-deoxy-2-(^18^F) fluoro-D-glucose (^18^FDG) as a Cerenkov radiation-emitting radionuclide and the photoinitiator, titanocene (TC) as a photosensitizer, we demonstrated that RaST successfully inhibited the progression of localized and disseminated tumors in mice. ^12,14^ The extensive cancer cell eradication in RaST-treated mice suggests the engagement of multiple cell death pathways beyond the direct effects of cytotoxic ROS alone.^12,13,15,16^

RaST utilizes a ROS generation process similar to PDT. A previous study suggested the involvement of inflammatory necrosis, which includes immunogenic damage-associated molecular patterns (DAMPs) in response to RaST.^14^ Another complementary cell death pathway caused by ROS-mediated enhancement of lipid peroxidation (ferroptosis) was recently implicated as a potential RaST-mediated cell death mechanism.^17^ Yet, these inflammatory and localized cell death pathways are insufficient to account for the downstream immunologic response, such as macrophage infiltration and apoptosis induction following RaST.^12^ However, apoptosis resistance is a hallmark of cancer evasion to chemotherapy,^18^ necessitating additional interventions that target extrinsic^19^ and intrinsic^20^ pathways to restore therapeutic effect. Recent studies have shown that apoptosis resistance can induce complementary necroptosis cell death.^21,22^ Although apoptosis and necroptosis are regulated cell death mechanisms, disparate signaling processes activate them. While apoptosis activates the executioner caspases through extrinsic or intrinsic pathways, initiating necroptosis involves activating receptor-interacting protein kinases 1 and 3 (RIPK1 and RIPK3). Unlike apoptosis, necroptosis promotes inflammation due to the release of DAMPs and pro-inflammatory cytokines. Therefore, therapies that activate necroptosis in response to apoptosis resistance or mediate the convergence of both pathways during treatment could improve cancer inhibition.

In this study, we sought to understand the dynamic response of cancer cells to RaST and the associated immunologic events. Our results show that RaST using ROS-regenerative titanium dioxide nanophotosensitizer coated with a tumor-targeting transferrin-titanocene complex (TiO_2_-TC-Tf) and ^18^FDG in cell cultures activates primarily the immunogenic apoptosis and necroptosis cell death pathways with secondary necrosis. This treatment was ineffective in inhibiting primary breast cancer progression and the associated metastatic disease in immune-deficient mice. In contrast, RaST achieved a significant complete response (CR) and a remarkable partial response (PR) and prevented metastasis to the lungs in immunocompetent tumor-bearing mice. Upregulation of RaST-induced immunogens such as necroptosis-associated cytokines and tumor antigens such as DAMPs was characteristic of positive response. Long-term surveillance of the CR mouse group using a tumor-avid agent, LS301,^23^ revealed the presence of residual tumors in the lymph nodes (LNs). An increase in the expression of negative immune regulating cytokine (TGF-β) and soluble immune checkpoints (sICP) in the CR group was insufficient to reverse tumor stasis. Translocation of TiO_2_ from the bones of mice shortly after treatment to the spleen and LNs in complete responders suggests the ROS-generating RaST component, TiO_2_, enabled immune cells to engage the residual disease and prevent metastatic spread and cancer relapse.

## RESULTS

### RaST activates immunogenic apoptosis and necroptosis cell death pathways

**Fig. 1|:**
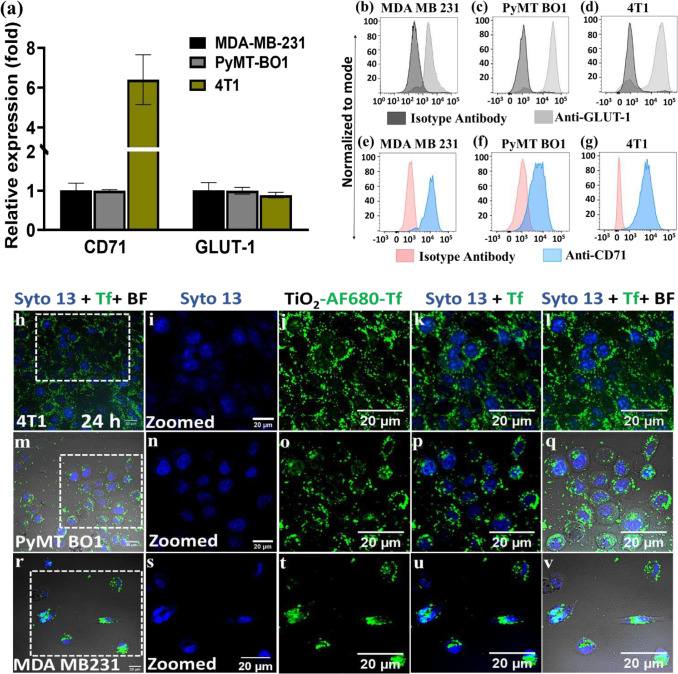
Cellular expression of target receptors and internalization of RaST components. (**a**) Quantitative analysis of CD71 and GLUT-1 mRNA expression using RT qPCR technique (n=6). (**b-d**) Quantitative analysis of CD71 and (**e-g**) GLUT-1 protein expression using flow cytometry analysis (n=3 independent sets). (**h-v**) Representative images of the internalization and distribution of Alexa Fluor 680-Tf-TO_2_ (25 μg/ 100 μl) in different breast cancer cell lines (n=3 independent sets). Dotted square insets are shown magnified in the images on the right. Scale bars = 20 μm, 1024 by 1024 aspect ratio, magnification 600 x with a confocal microscope.

An important goal of RaST is to maximize therapeutic effects on tumors while minimizing off-target toxicity. We accomplished this goal by orthogonally targeting transferrin (Tf) receptor^12^ and glucose transporter (GLUT-1)^24^ expressed on tumor cells with TiO_2_-TC-Tf and the radiopharmaceutical ^18^FDG, respectively. The convergence of these two RaST components generates ROS to induce cell death. Toward this objective, we first determined the expression of these biomarkers in MDA-MB231, 4T1, and PyMT-BO1 breast cancer cells. RT qPCR and quantitative flow cytometry showed all three cell types exhibited at least a 1-fold GLUT-1 mRNA increase above the background (Fig. 1a) and expressed the associated membrane proteins (Fig. 1b–g), respectively. Despite having similar mRNA levels, PyMT-B01 and 4T1 cells more efficiently translated GLUT-1 protein compared to MDA-MB-231 cells. Similarly, variability between Tf receptor (CD71) mRNA expression and protein translation was cell-dependent. Although 4T1 cells exhibited over 6-fold higher CD71 mRNA than background (Fig. 1a), all the cell lines efficiently translated the Tf receptor protein (Fig. 1e–g). These results support using any of the cell lines for orthogonal delivery of RaST components but also anticipate tumor model type-dependent response to RaST.

We next investigated TiO_2_-TC-Tf NP internalization in these cells using Alexa Fluor 680-Tf fluorescent analog (AF680Tf-TiO_2_). Confocal microscopy showed that all three cell lines adequately internalized the NPs (Fig. 1h–v), exhibiting the classic cytosolic punctate fluorescence expected for receptor-mediated endosomal trafficking after 24-hour incubation.^25^ Given that AF680-Tf may decouple from TiO_2_ NPs in cells, we explored an alternative technique to image the core NPs in cells. Alizarin red staining (ARS), which is widely used to determine calcium levels in cells, can also detect TiO_2_ NPs.^26,27^ However, the lack of specific binding of ARS to these metal ions has hampered their use in TiO_2_ cell microscopy. We found that ARS fluorescence in untreated cells was diffuse, consistent with the staining of mineral elements such as intracellular calcium.^28,29^ In contrast, distinctive punctate fluorescence was characteristic of intracellular binding of TiO_2_ NPs with ARS (**Supplementary Fig. 1 A-O**). Dark regions representing the NP’s intracellular distribution in the differential interference contrast (DIC) images correlated with the punctate ARS fluorescence pattern. This finding represents a new approach to rapidly determine TiO_2_ NP distribution in cells.

**Fig. 2|:**
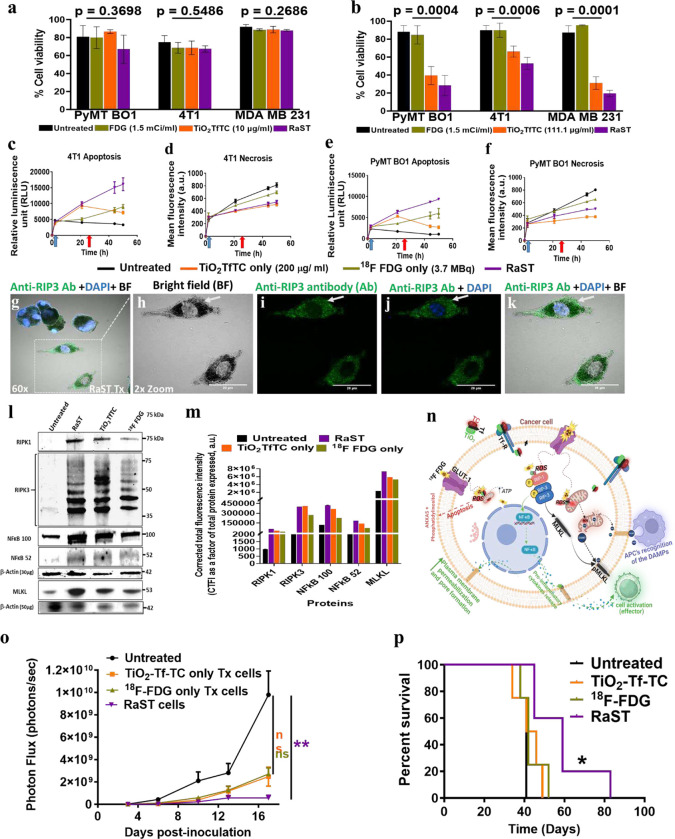
RaST-induced cell death mechanisms and host response to necroptosis challenge. **(a-b)** Dye exclusion cell viability test with Trypan blue dye post-RaST (^18^FDG + TiO_2_-TC-Tf) versus ^18^FDG alone or TiO2-Tc-Tf alone treatments in the three breast cancer cell lines using a (**a**) low (10.0 μg/ml) and (**b**) high (111.1 μg/ml) dose of TiO_2_-TC-Tf. There was mild to no cytotoxic effect for PyMT-BO1 (p = 0.3698), 4T1 (p =0.5486), and MDA-MB231 (p= 0.2686) in the low dose group. Groups treated with the higher concentration exhibited a significant cytotoxic effect in the RaST group compared to TiO_2_-TC-Tf, ^18^FDG, and the untreated groups. One-way ANOVA (non-parametric test) and Kruskal-Wallis multiple comparison tests for the PyMT-BO1 (p-value = 0.0004), 4T1 (p =0.0006), and MDA-MB231 (p= 0.0001) in the high dose showed significant cell type-dependent response. (**c-f)** Time-dependent cell death measurement using a parallel real-time apoptosis and necrosis assay on 4T1 and PyMT-BO1 breast cancer cells. Blue arrow is the initial background cell death reading at 2 h post-apoptosis and necrosis reagents addition, while the red arrow marks the ^18^FDG addition for RaST induction. (**g-k**) Confocal microscopy of RaST-treated PyMT-BO1 cells stained with anti-RIP3 Ab (green) and DAPI (blue) showing a strong positive RIP3 stain and morphological changes such as irregular membrane and pale nuclear stain at 48 h post-RaST. Scale bar 20 μm. (**l**) Western blot of necroptosis downstream signaling protein expression in the untreated, ^18^FDG, TiO_2_-TC-Tf, and RaST; RIP1, RIP3, NFkB 100 and 52 (30 μg/well), and MLKL (50 μg/well). (**m**) Quantitative analysis of the protein bands for each necroptosis-associated downstream protein using the manufacturer’s calculated molecular weights. (**n**) Schematic representation of the proposed cellular level necroptosis induction through a death receptor-independent pathway activated by RaST. (**o**) RaST-induced necroptosis challenge with pretreated PyMT-BO1 GFP Luc cells inoculated in mammary fat pads and tumor growth followed using BLI. (**p**) Kaplan Meier survival analysis. P-value = * ≤ 0.05, **≤ 0.01, and ***≤ 0.001. Blue and red arrows or triangles represent TiO_2_TC-Tf and ^18^FDG treatment, respectively.

We used a dye exclusion test to identify dead cells to understand how RaST and its components (TiO2-TC-Tf and 18FDG) impacted cell membrane integrity.^30^ At a fixed ^18^FDG activity of 55.5 MBq/ml (1.5 mCi/ml), response to RaST was both cell-type and TiO_2_-TC-Tf concentration-dependent (Fig 2a–b). Cells treated with low dose TiO_2_-TC-Tf (10.5 μg/ml) did not show any significant response to RaST or its NP component (Fig. 2a). However, the cytotoxic effects of RaST were evident at a higher TiO_2_-TC-Tf concentration of 111.1 μg/ml (Fig. 2b). By optimizing TiO_2_-TC-Tf and ^18^FDG doses, RaST could overcome the inherent resistance of the murine (4T1) and human (MDA-MB-231) triple-negative breast cancer cells to standard therapies. In addition to serving as a RaST component, TiO_2_-TC-Tf can exert a complementary therapeutic effect at high concentrations. Balancing this positive outcome via targeted RaST induction with potential sporadic side effects at high photosensitizer concentrations is necessary to minimize off-target toxicity during RaST.

Apoptosis and necrosis are two major cell death pathways in response to therapeutic interventions. The combination of real-time apoptosis (luminescence) and necrosis (fluorescence) assays allowed us to determine the contributions of these mechanisms to RaST’s cytotoxicity.^31^ We focused subsequent mechanistic studies on PyMT B01 and 4T1 cells, as RaST eradicated most MDA-MB-231 cells at high TiO_2_-TC-Tf concentrations. Time-dependent measurement of the interplay between necrosis and apoptosis showed that the untreated PyMT-BO1 and 4T1 cells died predominantly by necrosis rather than apoptosis (Fig. 2c–f). ^18^FDG treatment induced both apoptosis and necrosis cell death, significantly increasing the necrotic cell proportion over time. However, these cells recovered or maintained cell viability levels similar to the untreated cells 48 h after ^18^FDG treatment (Fig. 2 a,b). In TiO_2_-TC-Tf-treated cells, the initial dominance of apoptosis declined with time in both cell lines, indicating the inability of these NPs to sustain apoptosis after 20 h. This finding is consistent with other studies demonstrating how cancer cells mount apoptosis resistance in response to chemotherapy.^32–34^ Unlike ^18^FDG and TiO_2_-TC-Tf treated cells, RaST potentiated more durable apoptosis than necrosis over 48 h while sustaining a significant level of necrosis. These differential cell death patterns and rates suggest that RaST and its components mediate cell death predominantly via apoptosis induction.

Whereas the dye exclusion test showed significant RaST’s cytotoxic effect, these cells exhibited minimal response with ATP metabolic cell viability assay (**Supplementary Fig. 2C)**, suggesting that the dying cells retained some levels of metabolic activity.^35^ Assessment of RaST and TiO_2_-TC-Tf-treated cell morphology showed strongly adherent, blackish-discolored dying cells (Fig. 2g–k). The retention of metabolic activity, sustenance of secondary necrosis, and appearance of distorted cell morphology 48 h post-RaST point to a regulated form of necrosis such as necroptosis.

Cells can trigger necroptosis in response to cellular stress or apoptosis resistance by activating receptor-interacting protein kinases, RIPK-1 and RIPK-3.^36^ Subsequent activation and translocation of the executioner mixed-lineage kinase domain-like protein (MLKL) to plasma membranes disrupt the cell integrity, releasing DAMPs and pro-inflammatory cytokines.^37,38^ We found that RaST-treated cells, which showed low or no DAPI nuclei stains, exhibited a high expression of the RIPK-3 protein (green color) after staining with Alexa fluor 647 anti-RIPK-3 antibody (Fig. 2g–k). This led us to investigate the expression levels of relevant necroptosis downstream signaling proteins (RIPK-1, RIPK-3, nuclear factors kappa B (NFk_B_) 100 and 52 subunits, and MLKL) that occur through the death receptor pathway activated by tumor necrosis factor-alpha (TNF α). The higher resistance of 4T1 cells to RaST than PyMT-BO1 allowed us to harvest adequate apoptotic and necroptotic 4T1 cells for this study. Western blot revealed that RaST and its components activated repressed RIPK-1 and RIPK-3, enhancing the expression of the tightly regulated mature NF_K_B subunit, p52, and its inactive precursor subunits p100 (Fig. 2l–m). The extensive activation of major necroptosis signaling proteins during RaST supports the active participation of this cell death pathway. We found that RaST activated downstream NF_K_B and MLKL to enhance plasma membrane permeabilization and pore formation, respectively, followed by the release of DAMPs and cytokines (Fig. 2g–k, l–m, n, **Table 2)**.^37,39^ Comparison with a widely used apoptosis-inducing drug, doxorubicin, show that this chemotherapeutic agent was ineffective in activating RIPK-1 and RIPK-3 proteins (**Supplementary Fig. 3A-B**). Perhaps the inability of standard chemotherapeutics, such as doxorubicin, to induce necroptosis in apoptosis-resisting cells contributes to poor clinical outcomes in some cancer types. As new approaches to overcome doxorubicin resistance in breast cancer patients continue to emerge,^40,41^ combination therapy with RaST or other necroptosis-inducing drugs could improve treatment outcomes.

Induced immunogenic cell death is reversible, especially without immune cell engagement. Particularly, necroptosis-activated live/dead cells are expected to stimulate antitumor immune response in vivo and inhibit tumor progression. Unfortunately, there are no reliable and well-characterized 3D tumor and tumor-associated co-culture cell models to assess the efficacy of necroptosis-inducing drugs. To simulate this condition, we treated PyMT-BO1 GFP Luc breast cancer cells with TiO_2_-TC-Tf, ^18^FDG, and the associated RaST. About 50,000 cells from each treatment and untreated cells were implanted in the mammary fat pad (MFP) of immunocompetent C57BL/6 mice. The tumor growth was monitored by bioluminescence imaging (BLI) and caliper measurements. BLI shows that the RaST group significantly suppressed tumor progression (p=0.0038) compared to the insignificant outcomes with TiO_2_-TC-Tf (p= 0.2215) and ^18^FDG (p= 0.3912) relative to the untreated control (Fig. 2o), which correlated with data from caliper measurements (**Supplementary Fig. 5A-F**). The RaST-induced necroptosis model achieved a median survival of 59 days compared to 41, 44, and 42 days for the untreated, TiO_2_-TC-Tf, and ^18^FDG groups, respectively (Fig. 2P). The result demonstrates a new necroptosis challenge method for assessing the efficacy of necroptosis-inducing drugs to activate antitumor immune response. Fig. 2n illustrates the putative necroptosis cell death pathway and accompanying immune system stimulation.

### Convergence of TiO_2_-TC-Tf and ^18^FDG in tumors predicts favorable RaST response in vivo

**Fig. 3|:**
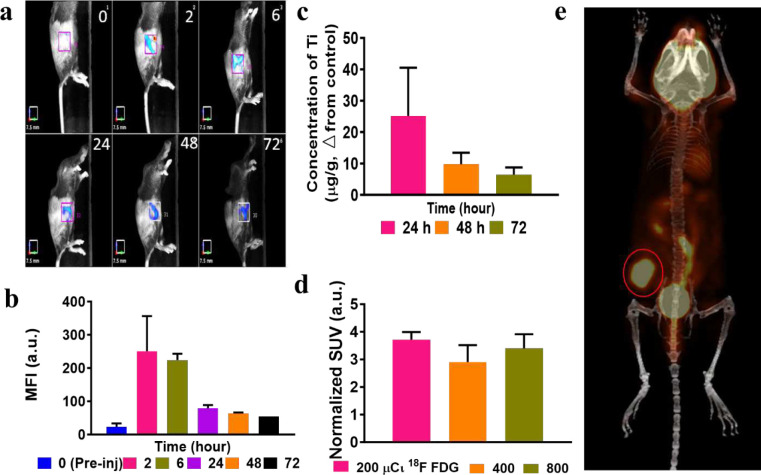
Biodistribution profile of the TiO_2_ NPs and ^18^FDG in C57BL/6 PyMT-BO1 GFP Luc MFP mouse model. (**a**) FMT imaging of tumor-bearing mice after tail vein injection of AF680-Tf-TiO_2_ (100 μg/ 100 μl) shows a fast accumulation of TiO_2_-Alexa fluor 680-Tf in the tumor at 2 h post-injection, followed by a gradual decrease in the mean fluorescence intensity over time (n=3/group). (**b**) Quantification of changes in the tumor mean fluorescence intensity at different time points. (**c**) ICP-MS analysis of titanium content (from TiO_2_-TC-Tf) in the tumor from 24 h post-injection (n=3/group). (**d**) SUV analysis of PET scans of the tumor site after 1 h showed no significant SUV difference in the three ^18^FDG doses used, favoring the use of the lowest activity for RaST (n=4/group). (**e**). Representative PET-CT image shows high tumor uptake of ^18^FDG (red circle), supporting the colocalization of both RaST components in the tumor (7.4 MBq/ 200 μCi, n=3/group).

RaST is more effective when the photosensitizer and Cerenkov radiation source selectively accumulate in tumors and concomitantly clear from the body via orthogonal excretion pathways. Toward this goal, we evaluated the distribution of the RaST components in an orthotopic PyMT-B01 MFP model in immunocompetent C57BL/6 mice. Fluorescence molecular tomography (FMT) and inductively coupled plasma mass spectrometry (ICP-MS) were used to determine TiO_2_-Alexa Fluor 680-Tf and TiO_2_-TC-Tf biodistribution, respectively. Quantitative FMT analysis indicates that 2 to 6 h post-injection of TiO_2_-Alexa Fluor 680-Tf is the optimal time range for maximum tumor accumulation based on Alexa Fluor 680 fluorescence (Fig. 3a–b). As shown by ICP-MS analysis (Fig. 3c), the Ti content in the tumor remained high for up to 24 h, suggesting that the Tf dissociated from the NPs during exocytosis, leaving the NPs in the cells. Although a significant decrease after 48 h was observed, tumor retention of the TiO_2_ NPs persisted up to 72 h post-injection, extending the window for initiating RaST in vivo. Positron emission tomography/computed tomography (PET/CT) of ^18^FDG (7.40 MBq/200 μCі) distribution confirmed its high tumor uptake within 2 h post-injection (Fig. 3e), demonstrating the colocalization of both RaST components in this tumor model is achievable. To optimize RaST effect, we evaluated whether higher ^18^FDG doses (14.8 MBq/400 μCі and 29.6 MBq/800 μCі) would enhance tumor accumulation. Standardized uptake value (SUV) analysis shows that the tumor accumulation was less dependent on the radiopharmaceutical dose (Fig. 3d). Therefore, we used the lowest radioactivity dose (200 μCi) for RaST to minimize radionuclide-induced toxicity to healthy tissues.

### RaST inhibits tumor progression and prevents lung metastasis in immunocompetent mice

**Fig. 4|:**
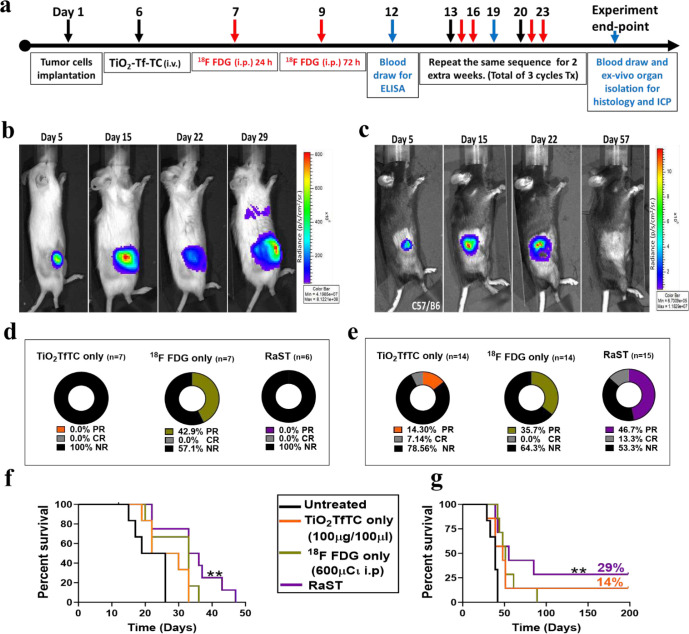
Assessment of cumulative treatment response using the immune-related response criteria. (**a**) Schematic for the in vivo RaST study. (**b**) Representative BLI images of tumor progression on different days post-treatment using immunocompromised NSG mice inoculated in MFP with PyMT-BO1PyMT-BO1 cells. (**c**) Representative BLI images of tumor progression on different days post-treatment using immunocompetent C57BL/6 mice inoculated in MFP with PyMT-BO1 cells. (**d-e**) Cumulative RaST response assessment using the immune-related response criteria (irRC) categorized into no-response or progressive disease (NR), partial response (PR) and complete response (CR) in (**d**) NSG PyMT-BO1orthotopic MFP model: Untreated n=3, TiO_2_-TC-Tf n=5, ^18^FDG only n=6 and RaST n=6, and (**e**) C57BL/6 PyMT-BO1 orthotopic MFP model, Group A: Untreated n=6, TiO_2_-TC-Tf n=7, ^18^FDG only n=7 and RaST n=8 and Group B: Untreated n=6, TiO_2_-TC-Tf n=7, ^18^FDG only n=7 and RaST n=7. **(f and g)** Th**e** Kaplan-Meier survival plots of C57BL/6 mice inoculated with (**f**) 1.0 × 10^5^ [Group A] and (**g**) 5.0 × 10^4^ [Group B] PyMT-BO1 GFP Luc cells. A significant survival difference and improved overall survival were observed in the Group B cohorts.

The predominant immunogenic cell death pathway observed in the in vitro studies suggests that RaST could leverage the immune system to exert a durable treatment response in mice with intact immune components. We tested this hypothesis by performing an in vivo RaST study with severe immunodeficient NOD SCID (NSG) and fully immunocompetent C57BL/6 mice. Previous studies have demonstrated that the Matrigel tumor implantation model recapitulates human stroma and some growth factors that prime the microenvironment for aggressive tumor progression in mice.^42,43^ Co-implanting PyMT-BO1 GFP Luc cells and Matrigel in the MFP of the NSG and C57BL/6 mice produced an aggressive orthotopic breast cancer model. The treatment design using TiO_2_-TC-Tf, ^18^FDG, and RaST is summarized in Fig. 4a. In addition to inoculating 1.0 × 10^5^ tumor cells (Group A) in both mouse models, we also implanted 5.0 × 10^4^ tumor cells (Group B) in the C57BL/6 mice to compare the effects of different starting tumor densities on RaST response.^44^ BLI, caliper, and weight measurements were used to determine the tumor growth pattern and response to treatment. Applying the immune-related response criteria^45^ allowed us to classify the evolving pattern of response into three categories: (1) no response (NR), partial response (PR, tumor volume ≤ 500 mm^3^ for Group A and ≤ 250 mm^3^ for Group B) and complete response (CR, no visible, palpable or BLI detectable tumor cells).

In the NSG PyMT-BO1 Luc model, all the treated and untreated mice developed aggressive tumors, which spread to the lungs (Fig. 4b). Although the ^18^FDG-treated mice exhibited partial response (42%) compared to the TiO_2_-TC-Tf and RaST groups (Fig. 4d, **Supplementary Fig. 5A-B**), all the NSG mice were euthanized when they reached primary tumor growth endpoint of 2 cm or confirmed moribund within 29 days. Perhaps the increased tumor burden associated with the lung metastasis accelerated moribund state in some of these mice. This result highlights the inefficiency of RaST in inhibiting tumor growth in immunodeficient mice. Both RaST and its individual treatment components were unable to prevent the spread of disease in this breast cancer model. In contrast to the NSG mice, the PyMT-BO1 Luc tumors responded to RaST in the C57BL/6 cancer model (Fig. 4c, **Supplementary Fig. 5C-D**). Aggregating the data for Groups A and B cohorts initiated with different cancer cell densities (see above) shows that RaST achieved 47% PR and 13% CR compared to the other treatment groups in this aggressive tumor model (Fig. 4 e).

Similar to the NSG mice, ^18^FDG achieved a significant PR without any CR, corroborating the reversible effects of ^18^FDG in the cell culture study. Analysis of the survival rate for the two separate groups shows that the Group A mice (Fig. 4f, **Supplementary Fig. 5C-D**) reached the 2 cm endpoint much faster than Group B (Fig. 4g, **Supplementary Fig. 5E-F**), regardless of the treatment module. The dense tumor cell growth from days 15 to 22 decreased the BLI signal, probably due to blood vessel occlusion and an increase in the proportion of dead cells. The Kaplan-Meier survival plot, extracted from the onset of BLI dampening, demonstrated a significant difference between each treatment based on the Log-rank (Mantel-Cox) Test p-value of 0.0038 and 0.0040 for Groups A and B, respectively. Compared to 22.5 days median survival for the untreated cohort in Group A, TiO_2_-TC-Tf, ^18^FDG, and RaST treatments prolonged the median survival to 26, 33, and 35 days, respectively. A similar trend was observed for Group B, with corresponding median survivals of 48, 51, and 55 days. Remarkably, RaST achieved a CR of 29% in Group B, suggesting that the treatment could efficiently eradicate small tumors. This conclusion aligns with the ability of RaST to inhibit lung metastasis in the C57BL/6 compared to the persistence of the disseminated disease in the TiO_2_-TC-Tf and ^18^FDG treated groups.

### RaST recruits myeloid and lymphoid tissue immune cells to inhibit tumor progression

**Fig. 5|:**
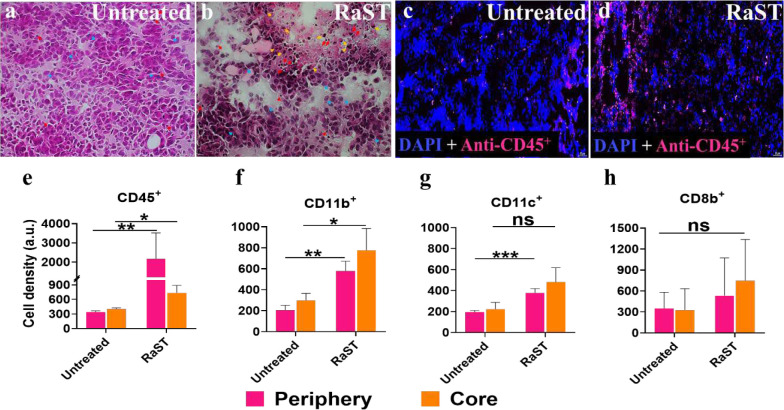
Immune cell infiltration into tumors in response to RaST. **(a)** H&E staining of untreated PyMT-BO1 tumor tissue from C57BL/6 mice. (**b**) H&E staining of RaST-treated PyMT-BO1 tumor tissue from C57BL/6 mice inoculated with low density (5.0 × 10^4^) tumor cells on day 15 (40x, representative images from n=5 images per group). **(c)** Representative immunofluorescence image of untreated tumor tissue stained with anti-CD45 Ab on day 15**. (d)** Representative immunofluorescence image of RaST-treated tumor tissue stained with anti-CD45 Ab on day 15**. (e-f)** Quantitative analysis of the immunofluorescence-stained tumor tissue from untreated and RaST group for (e) CD45^+^, p-value periphery 0.1957 and core 0.1264, (**F**) CD11b^+^, (p-value periphery 0.0017 and core 0.0289), (g) CD11c^+^, (p-value periphery 0.0013 and core 0.0649), and (h) CD8b^+^, (p-value periphery 0.1957 and core 0.1264) biomarkers at the periphery (pink) and core (yellow) regions. Image quantitative analysis used 3–5 hot spots from 3 representative mice tumor tissues per group and presented as mean ± SEM followed by a simple t-Test analysis, p-value <0.0001 ****, <0.001***, <0.01 **, <0.05 *.

Differences in the tumor response to RaST between the NSG and C57BL/6 PyMT-BO1 models suggest that adequate immunologic induction suppressed tumor growth, facilitated tumor eradication, prolonged survival, and inhibited metastasis. The survival study identified a group of three mice with CR in the RaST and TiO_2_-TC-Tf treated groups. BLI at different intervals was used to rule out tumor relapse throughout the 167-day surveillance period for CR mice. This allowed us to assess changes in immunologic response over a wide range of time during and after treatment. The histology of the tumor tissues was obtained on day 15 or 19 post-inoculation. The untreated tumor demonstrated scattered necrotic tumor cells with dense eosinophilic cytoplasm (blue arrows) with very rare tumor-infiltrative immune cells (red arrows) at the center and periphery of the mass (Fig. 5a). The RaST-treated tumor displayed areas of necrosis, degenerative tumor cells (blue arrows), and apoptotic bodies (yellow arrows). Increased tumor-infiltrative immune cells (red arrows) were also noted (Fig. 5b).

Our results demonstrate that RaST recruited tumor-infiltrating immune cells into tumor tissue obtained from PyMT-BO1 tumor-bearing C57BL/6 mice. We used multiple assays to identify potential effector immune cells activated by RaST-induced immunogenic cell death. Using immunofluorescence techniques, we determined the number of white blood cells and some effector immune cell surface biomarkers. These include CD45 (common leukocyte marker), CD11b (myeloid-lineage leukocyte marker), CD11c (lymphoid tissue dendritic cell marker), and CD8b (cytotoxic T cell marker; Fig. 5e–h). The untreated group exhibited sparse CD45^+^ cells, indicating a limited amount of tumor-infiltrative immune cells (Fig. 5c). In contrast, a significant number of CD45^+^ cells were in the tumor of the RaST-treated group compared to the untreated control (Fig. 5d), with a sizable number in the periphery (p-value 0.0077) than the core (p-value 0.0461) of the tumor (Fig. 5e). The presence of intact ducts and areas of dense tumor cells (yellow arrow) infiltrated by CD45^+^ immune cells in the RaST-treated group highlights a spatiotemporal distribution of the immune cells in these regions (**Supplementary Fig. 6 B-C**). Normal MFP architecture, such as the mammary adipocyte pockets (red arrow) and ducts (white arrow), was unaffected, indicating that RaST could spare surrounding healthy tissue. The immunofluorescence stains showed an increased CD11b^+^ cell density at the tumor periphery and core in the RaST compared to the untreated mice (Fig. 5f). The dendritic cell population (CD11c^+^) in the RaST group also increased compared to the control. Although the spatial distribution of these cells in the periphery of the RaST and untreated groups was different, the core regions were similar (Fig. 5g). Collectively, these results demonstrate that RaST recruited a higher number of CD11b^+^ and CD11c^+^ tumor-infiltrating immune cells than the marginal CD8b^+^ cells (Fig. 5h), indicating the dominance of innate than adaptive immune response.

### Necroptosis-associated cytokines signaling mediate RaST-induced immunomodulation

**Fig. 6|:**
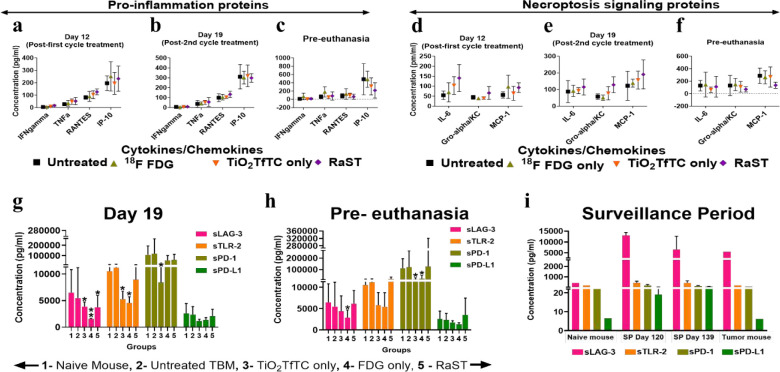
Immunomodulation profile during and after treatment. **(a-f)** Time-dependent immuno-ELISA assay analysis of plasma cytokines associated with (**a-c**) pro-inflammation and (**d-f**) necroptosis antitumor effect after the first treatment cycle (Day 12 post-implantation), post-second treatment cycle (Day 19 post-implantation), and pre-euthanasia (endpoint at 2 cm tumor size) using TiO_2_-TC-Tf**,** ^18^FDG, and RaST. **(g-h)** Time-dependent immuno-ELISA assay of plasma soluble immune checkpoint proteins **(g)** post-second treatment cycle (Day 19 post-implantation), (**h**) pre-euthanasia, and (**i**) surveillance periods using the human IO kit (n=3 to 7 samples per group), mean ± SD.

The dynamic assessment of tumor response of individual mice used in this study showed adequate response during therapy, followed by a gradual relapse post-treatment (**Supplementary Fig. 5A-F**). This trend is consistent with published data for other therapeutic interventions.^44^ Cancer cells reprogram to suppress immune response or enhance immune exhaustion for therapies that exert their effects via immunogenic cell death mechanisms. Therefore, we explored the effects of RaST on cytokines, chemokines, and soluble immune checkpoint proteins on days 12, day 19 post-implantation, and pre-euthanasia (measurements taken when animals reached the study endpoint). Plasma samples collected from mice at different time points were analyzed with a 28-Plex cytokine/chemokine panel and 4-panel immune checkpoint proteins immuno-assay. Untreated mice were used as a baseline for identifying relevant cytokines potentiated from each treatment module in the Group B cohort. The data was first analyzed using a heat-map with annotation of the differences: “+” represents RaST-induced cytokines/chemokines amount above the ^18^FDG level, and “=“ identifies cytokines/chemokines in the RaST and ^18^FDG cohorts above the untreated group concentration. We identified 24 and 4 RaST-induced “+” and “=“ cytokines, respectively, at the end of the first treatment cycle (**Supplementary Fig. 7A-C**). After the second treatment cycle, no new cytokines were identified, but a few transposed between the “+” and “= “groups, yielding a total of 25 “+” and 3 “=“ cytokines. Data from the pre-euthanasia period identified only 3 “+” (CRP, IL-12p70, and TGF-β) and 6 “=” (GM-CSF, IFNγ, IL-4, IL-5, IL-27, and TNFα) cytokines. These results show that RaST-induced high expression of positive immunoregulatory cytokines, C-reactive proteins (CRP), and interleukin 12 (IL-12p70) at the pre-euthanasia stage. With an interest in the inflammatory cytokines exhibiting antitumor response and necroptotic induction, we focused on IFN-gamma, TNF alpha, CCL5 (RANTES), CXCL10 IP-10, IL-6, CXCL1 (Groalpha KC), and CCL2 (MCP1) cytokines (Fig. 6a–f).^37^ On day 12 and day 19, RaST-induced inflammation and necroptosis-associated cytokine and chemokine levels were moderately higher than the other treatment groups. A similar analysis at pre-euthanasia shows that the concentrations of these cytokines and chemokines were either equal or lower than the other treatment groups. This result suggests that systemic inflammatory and necroptosis-inducing cytokines and chemokines were primarily upregulated during the early phase of RaST compared to the untreated, TiO_2_, and ^18^FDG treated groups.

The observed RaST-associated antitumor immune response could be attributed to its stimulatory effect on immune cells and tumor antigen presentation.^8^ We used the quantitative label-free protein LC-MS/MS technique to assess the PyMT-BO1 tumor-associated antigens status on day 19 post-implantation compared to the untreated control. The proteomics analysis revealed that RaST depleted the expression of heat shock protein 60 (HSP60) and calreticulin (**Supplementary Table 1A-B**). In addition to its multifunctional roles as a molecular chaperone, previous studies have demonstrated that HSP60 can promote cancer survival and apoptosis resistance.^46^ Considering that apoptosis resistance activates necroptosis, RaST could overcome this resistance via necroptosis induction. The tumor-associated antigen, calreticulin, is primarily located in the endoplasmic reticulum, but its translocation to the extracellular membrane triggers an immune response.^47,48^ Eradication of these antigen-positive cells or a progression-free state with minimal immune surveillance could account for the observed decrease for both antigens.

Whereas the immune system utilizes acute inflammation and adaptive immunity to control or eliminate cancers,^49–53^ chronic inflammation promotes tumor development. It also allows tumors to select for cancer cells with lower immunogenicity through interactions with checkpoint receptors and ligands. To further investigate the immunogenic response of cancer to RaST, we examined the expression of four soluble immune checkpoint receptors and ligands (sICRL) using the human immuno-oncology (I-O) kit on day 19 and pre-euthanasia period with plasma samples from the Group A cohort and during the surveillance period on day 120 and 139 of the three tumor-eradicated mice in the Group B cohort (Fig. 6 g–i). We found that the mean concentrations of the soluble lymphocyte activation gene 3 (sLAG-3), Toll-like receptor 2 (sTLR-2), programmed cell death-1 (sPD-1), and programmed death-ligand 1 (sPD-L1) receptor proteins from naïve C57BL/6 mice were comparable to the untreated tumor-bearing group on day 19 and pre-euthanasia period (Fig. 6g–h). However, all the treatment groups suppressed the mean sLAG-3 expression on day 19. RaST and its components also suppressed sTLR-2, a positive sICRL, on day 19 (Fig. 6g). In all cases, RaST effect on these sICRL was marginal. Extensive data variability was expected as samples from responders and non-responders were used in the analysis. In agreement with the tumor burden response, this result suggests that suppression of sLAG-3 at the onset of RaST enhanced antitumor immune and cytotoxic effects.

### Dynamic redistribution of TiO_2_ in lymphoid organs prevents metastasis

**Fig. 7|:**
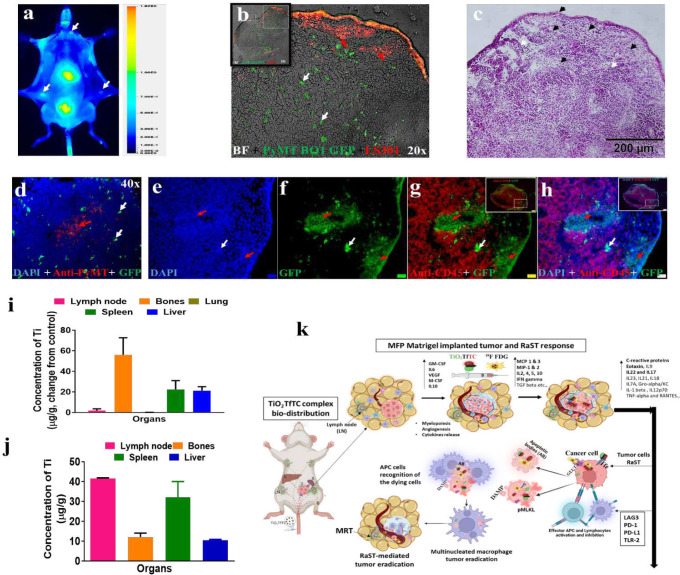
In vivo delineation and characterization of residual tumor cells in the LNs and the ROS-regenerative nanoparticle biodistribution. **(a**) In situ skin-exposed mouse showing multiple metastatic LNs identified by the LS301 (60μM, 100 μl; white arrow) retained at 24 h, a known characteristic of cancer and cancer-associated cells (representative image from 3 mice). (**b)** Fluorescence imaging of the sentinel and contralateral LNs for LS301-targeted cells using an epi-fluorescence microscope (Olympus) 40x lens and Cy7 channel filter. **(c)** H&E-stained LN tissue imaging confirms locations of the tumor cells (black arrow) and resident immune cells (white arrow), 20x merged images. (**d-h**) Representative immunofluorescence staining of LN tissues for (**d**) PyMT with anti-PyMT antibody and (**e-h**) white blood cells with CD45^+^ antibody. Scale bar for inset = 200 μm and zoomed images = 20 μm. (**i-j)** ICP-MS analysis of some major immune organs to determine Ti content from TiO_2_-TC-Tf NPs (**i**) after euthanasia for PR mice and (**j**) day 192 surveillance endpoint (post-RaST) in the CR mice (n=3, mean ± SD for each PR and CR groups). Bones include femurs, tibia, and fibula. **(k)** Schematic representation of factors and pathways that impact tumor progression and immunogenic response of tumors to RaST.

Some of the treated mice achieved CR without palpable lesions or BLI-detectable tumors during the surveillance period. These mice allowed us to compare changes in the sICRL levels at later time points (day 120 and day 139 post-implantation) with the early treatment cohorts. RaST-treated CR mice showed a remarkable increase in the mean concentrations of negative regulators of the immune system, sLAG-3, and sPD-L1, compared to the naïve and untreated tumor-bearing mice (Fig. 6i). The upregulation of these tumor-promoting sICRL in the CR mice could indicate the presence of minimal residual disseminated tumor (MRDT). Previously, we reported that a new pan-cancer near-infrared fluorescent imaging agent, LS301, could be used to detect small tumors through a signal amplification mechanism.^23^ To screen for MRDT, we injected LS301 via tail vein in the animals, followed by whole-body fluorescence imaging 24 h post-administration. Positive fluorescence signals were visualized in multiple LNs, including the right inguinal sentinel, contralateral inguinal, and axillary LNs (Fig. 7a). Histologic assessment of these LNs identified the green fluorescence of PyMT-BO1 GFP tumor cells, some of which co-localized with LS301 fluorescence (red arrow) in the periphery (Fig. 7b). In addition to some cancer cells with high LS301 uptake (red) but low GFP signal (green), multiple isolated single or clustered tumor cells (white arrow) exhibiting strong GFP but weak LS301 fluorescence were observed (Fig. 7b; **Supplementary Fig. 8A-C**). This fluorescence pattern conforms with LS301 mechanism of action. The expression of activated annexin A2 (pANXA2), a target of this agent, is low in metabolically less active cancer cells.

Conversely, cancer cells upregulate pANXA2 in surrounding cancer-associated fibroblasts and tumor-associated macrophages, enhancing the ability of LS301 to highlight regions of microscopic tumors. For example, the distinct pale cytosolic eosin stain with weak to moderately stained large nuclei around the capsular, subcapsular, and trabecular LN region correlated with the location of PyMT-BO1 GFP MRDT cells exhibiting low GFP fluorescence (Fig. 7c, black arrowhead). In contrast, uninvolved LN cells displayed the expected small-sized, dark purple nuclei with low to no cytoplasmic eosin staining (white arrowhead). Anti-PyMT immunofluorescence confirmed that the clustered cells exhibiting low GFP fluorescence are disseminated PyMT-BO1 GFP cells (Fig. 7d, red arrow). However, this antibody poorly stained the multiple isolated single or nodular cells with strong GFP fluorescence (Fig.7d, white arrow). The cells with strong nodular GFP fluorescence could be tumor cells with a different morphology than the clustered PyMT-BO1 cells with weak GFP signals.

Using anti-CD45^+^ antibody staining for resident white blood cells in the LNs, we show that the micro-metastatic and the clustered PyMT-BO1 GFP cells with strong and low GFP signals, respectively, are not antigen-presenting cells (APCs, Fig. 7e–h). Compared to the surrounding CD45^+^ immune cells, the GFP cells showed a large, intense DAPI (blue) nuclear stain (red and white arrows), a hallmark of cancer cells. Our results suggest that cells with strong GFP fluorescence could be transformed cells in a dormant or senescence state. They are sparsely distributed away from the clustered PyMT-BO1-GFP cells with low fluorescence (Fig. 7d, red arrow). Future studies will confirm the identity of these GFP cell phenotypes.

All the CR mice in this study were from the group that received RaST or TiO_2_ NP treatments. Despite harboring MRDT in the LNs, the tumors did not relapse or progress to metastatic sites such as the lungs. This observation prompted us to determine if the ROS-regenerative TiO_2_ distribution could facilitate immune cell activation. ICP-MS analysis of RaST-treated and untreated C57BL/6 mice from the low cancer cell density model (Group B) showed a high accumulation of Ti in bones (58 μg/g), spleen (23 μg/g), and the liver (20 μg/g), with moderate uptake in the LNs (3 μg/g), referenced to the untreated mice at pre-euthanasia (Fig. 7i). The high retention of TiO_2_ in lymphoid organs increases the probability of resident immune cells, such as phagocytic cells, to recognize the NPs and induce immunogenic response.^15,54^ We extended this analysis to CR mice on day 192 post-RaST. Our result shows a remarkable translocation of TiO_2_ from bones to the LNs and an increase in the spleen of CR mice (Fig. 7j). This finding implies the active engagement of TiO_2_ with immune cells to prevent the migration of MRDT cells to sites of metastasis, perhaps through TiO_2_ regenerative ROS induction.^55–57^

## Discussion

RaST requires selective cellular uptake of the NPs and ^18^FDG to achieve sustainable cell death. We established that all the cell lines used in this work expressed the target Tf and GLUT-1 proteins for the orthogonal delivery of RaST components. This study demonstrated that the punctate fluorescence pattern of ARS in cells incubated with TiO_2_ NPs is a unique feature that distinguishes it from the diffuse background fluorescence of intrinsic mineral elements. Compared to the arduous steps of labeling NPs with ARS dye,^26,27^ which could interfere with Tf coating and alter their intracellular distribution, our ARS staining approach preserves the integrity of the TiO_2_-TC-Tf NPs, and allows rapid determination of NP distribution in cells (**Supplementary Fig. 1A-O**). Future studies will explore the potential application of this method in determining the dynamic uptake of inorganic NPs that bind ARS.

The short doubling time (11–37 h) of the aggressive breast cancer cells used in this study facilitated their recovery and propagation within 4 days after in vitro treatment (**Supplementary Fig. 2A-B**). Thus, induction of durable cellular injuries by RaST-induced cytotoxic ROS would require the generation of high levels of cytotoxic ROS in most cancer cells while preventing intrinsic survival mechanisms.^58,59^ A High ROS threshold is practically not achievable in most solid tumors without loss of selectivity for cancer cells. Many conventional drugs are known to induce DNA methylation or histone deacetylation-dependent loss of RIP3, resulting in chemotherapy-resistant cancer cells such as those used in this study.^60^ In contrast to chemotherapeutics such as doxorubicin, RaST can overcome this resistance by upregulating the expression of RIP1 and RIP3 to pivot into an alternative necroptosis cell death pathway. Interaction of RIP 1 and 3 with the matured NF-_K_B p52 is known to form a heterodimer with the Rel NF-_K_B. Subsequent translocation of the ensuing active NF-_K_B complex into the nucleus and DNA binding stimulates the transcription of cytokines to potentiate MLKL membrane permeabilization or pore formation.^61^ The significant DAMP release and slow cancer cell death in the absence of immune cells *in vitro* supports RaST-mediated immunogenic cell death mechanism and highlights the potential of using RaST in combination therapies.

In line with the in vitro findings, the in vivo study reveals that RaST, and its components exerted a robust tumor response in immunocompetent mice but failed to inhibit breast cancer progression in severely immunocompromised mice. The rapid tumor growth and metastasis in all the immune-deficient mice indicate active immune system involvement during RaST. These observations appear to contrast with previous studies where RaST inhibited the progression of both hematological^12^ and solid^14^ tumors in immunodeficient mouse models. Although the immune-deficient mice used in this (NSG) and the previous (homozygous athymic nude and Fox Chase SCID Beige) studies lack functional T cells and defective B cells, they retain a significant level of innate immunity, evidenced by macrophage infiltration and denuding of tumor tissue in these RaST treated models.^12^ In the absence of adaptive immunity, RaST initially achieved significant remission, but the cancer ultimately relapsed, analogous to standard chemotherapy. Given the similarity of the immunodeficient mice used in these studies, we attribute the differential response observed in our present research to differences in the cancer model employed.

The Matrigel-supported orthotopic mammary fat tumor model provides an environment with adequate extracellular matrix proteins and growth factors that select for aggressive tumor growth similar to immune-rich human tumors.^42,43^ Formation of this diffusion-limited cold tumor environment confines inorganic NPs such as TiO_2_ to distinct areas in the tumor tissue. Under these conditions, RaST was expected to exert its effect via non-immunogenic apoptosis and necrosis on cells close to RaST components. Indeed, the BLI signals and tumor volumes NSG mice undergoing treatment fluctuated tremendously before euthanasia (**Supplementary Fig. 5A-B**). Perhaps the high glucose metabolic activity of these tumors accounts for the observed initial partial response of NSG mice treated with ^18^FDG alone. Without a complementary adjuvant immunologic response, RaST and its individual component were unable to sustain tumor inhibition in this aggressive cancer model.

Although RaST can inhibit different-sized tumors, it is more effective in eradicating smaller lesions. Using two models of late (Group A) and early (Group B) stage cancer, RaST achieved 29% CR in the Group B cohort compared to the only PR found in Group A. Further, the treatment prevented lung metastasis in both groups. These rare positive outcomes with the Matrigel MFP PyMT-BO1 tumor model probably occurred through RaST-induced immunopotentiation. Previous reports demonstrated that some human tumors exhibit continuous myelopoiesis during disease progression.^62–64^ This process produces a variety of myeloid cells, including the myeloid-derived suppressor cells that infiltrate lymphoid organs and growing tumors to suppress or enhance tumor progression. Indeed, histologic analysis of treated tumor tissues shows evidence of denuded areas filled with dead tumor cells and CD45^+^ white blood cells, indicative of adequate immune cell recruitment during RaST (Fig. 5b). CD45^+^ is a common leukocyte antigen expressed by many hematopoietic cells that can serve as positive or negative regulators of tumor progression. Other studies with similar tumor models demonstrated higher tumor mass infiltration by bone marrow-derived CD11b^+^ Gr-1^int/dull^ Ly-6C^hi^ macrophages than CD11b^+^Gr-1^hi^Ly-6C^int^ neutrophils, suggesting that CD11b^+^ Gr-1^int/dull^ Ly-6C^hi^ macrophages could dominate the tumor microenvironments in these tumors.^65^ In accordance with these reports, we identified statistically significant high levels of CD11b^+^ and CD11c^+^ cells but moderately higher density of CD8b^+^ cytotoxic T cells compared to the untreated cohort in our immune-competent tumor model. These results indicate the upregulated necroptosis and inflammatory antitumor immune-activating cytokines expressed during therapy (days 12 and 19 post-implantation) favored the recruitment of antigen-presenting innate cells (CD11b^+^ macrophage and CD11c^+^ dendritic cells) over adaptive immune cells (CD8b^+^).

The high tumor infiltration of CD45^+^ immune cells in the RaST group could be attributed to the upregulation of 25 cytokines before PR mice became unresponsive. Dynamic changes in the expression levels of these cytokines were observed over time, reflecting their complex roles in immunogenic cancer cell death. Notable among these are the high levels of the monocyte chemoattractant protein-1 (MCP-1/CCL2) and transforming growth factor-beta 1 (TGFβ) on day 19. MCP-1, a chemoattractant secreted by some immune cells, attracts monocytes, dendritic cells, basophils, eosinophils, and memory T cells,^66^ making it a potent recruiter of innate immune cells. TGF-β1 is a multifunctional white blood cell-secreted protein that could function as a negative regulator of immune response. Upon activation and release of the TGF-β from the surface of macrophages, the naive T cells differentiate into T regulatory cells (Treg). These Tregs participate in the regulation of apoptosis induction, inhibition of monocyte and macrophage cytokines secretion (e.g., MCP-1)^67^ and NF-KB regulated cytokines (e.g., IL-1 and TNF-α).^68^ The high expression of TGF-β1 before RaST group became non-responsive implicates it as a major negative regulator cytokine for suppressing antitumor immune response.^69^ Other cytokines upregulated during therapy that persisted up to the pre-euthanasia period include IL-12p70 and CRP. IL-12p70 is the heterodimer (p35 and p40 subunits) form of the IL-12 cytokine secreted by dendritic, macrophage, neutrophils, and B cells in response to antigen stimulation.^70^ Their release induces naïve T cells to differentiate into Th1 cells. APC cells bind and activate Th1, producing cytokines such as IFN-γ and TNF-α.^71,72^ CRP, a liver acute phase reactant and a pattern recognition protein, is secreted in response to releasing pro-inflammatory cytokines such as IL-6, IL-1, TGF-β, and TNF-α in inflamed tissues.^73,74^ Although CRP has a short half-life of 4 to 7 h in blood,^75^ it persisted in the RaST group, suggesting its vital role in the innate immune response. As an opsonin, CRP could bind TiO_2_-TC-Tf NPs and dying cells to initiate complement activation and macrophage phagocytosis. The immune-ELISA data showed high expression of monocyte chemotactic proteins (MCP-3/CCL7, MCP-1/CCL2) and macrophage inflammatory proteins (MIP-1/CCL3 and MIP-2/CXCL2), suggesting a high level of monocyte recruitment.

We also found that RaST enhanced the expression of pro-inflammatory cytokines, IL-22 and IL-17. CD4^+^ Th17, NK cells, and T cells are known to co-express IL-22 and IL-17 following the stimulation of IL-6, IL-21 (not assayed in this 28plex assay) and IL-23. These cytokines bind receptors on non-hematopoietic cells to activate intracellular kinases (Janus kinase, JAK; tyrosine kinase, TyK2; and mitogen-activated protein kinase, MAPK) and transcription factors such as the signal transducer and activation of transcription 3 (STAT3) to support cell growth, inflammation, or apoptosis.^76^ Major cytokines secreted by lymphocytes such as IL-2, INF-γ, TNFα, IL-4, and IL-5 were among the upregulated cytokines in plasma, suggesting that T and B cells’ adaptive immunity was activated. However, some of them decreased in the interval between post-second cycle treatment and pre-euthanasia. These dynamic changes in cytokine expression are necessary to modulate the activity of immune cells. In particular, some cytokines and immune checkpoint proteins inactivate T and B cells in the tumor microenvironment during their lifespan.^77,78^ For instance, Th2 and Th1 T cell subsets secrete IL10 cytokine and INF-γ to inhibit CD8^+^ and CD4^+^ B cells activation, respectively. These inhibitory activities also allow anti-inflammatory cytokines such as IL-10, TGFβ, and IL-1β identified in this study to heal and restore tissue integrity after solid tumor eradication. A notable switch between PR and CR mice is the inversion of sLAG-3 expression. The significant decrease of this protein in treated cohorts compared to the naïve and untreated groups during therapy (day 19) correlated with the initial treatment response. Before euthanasia, the protein level was restored in the RaST group, demonstrating that the inhibitory role of LAG-3 accelerated tumor progression and subsequent death of these mice. However, the persistence of sLAG-3 in CR with no visible sign of residual tumors was surprising. While we did not determine if RaST-induced LAG-3 expression played a dual role in this study, co-habitation with the LN immune cells probably resulted in sustained immune tolerance and exhaustion. The redistribution of TiO_2_ NPs from bones and liver to the LNs and spleen of these CRs with no obvious evidence of metastatic lesion invokes the possibility of continuous engagement of the ROS-generating NPs with immune cells to suppress the activation and migration of MRDT cells.

In conclusion, this study shows that RaST deploys immunogenic apoptosis and necroptosis cell death pathways to exert durable tumor inhibition in an aggressive model of breast cancer, as summarized in Fig. 7k. The complementary nature of these cell death mechanisms could be exploited to achieve long cancer remission. While RaST suppressed negative immune regulators at the early stages of the treatment, cancer cells reprogrammed and re-established these proteins and factors to accelerate tumor progression. Nonetheless, none of the RaST group developed lung metastasis. Despite the apparent complete remission for the CR mice, a small peptide-dye conjugate, LS301, revealed the presence of MRDT in the LNs through a signal amplification mechanism, uncovering a new approach to identifying residual disease. Translocation of the ROS-regenerative NPs from the bones and liver to the spleen and LNs kept the MRDT in check, preventing cancer relapse. With the availability of checkpoint inhibitors and other drugs that act via alternative mechanisms, strategic combination therapy with RaST could improve cancer remission and minimize relapse.

## Method

### Cell culture

4T1 murine adenocarcinoma (HER2^−^, ER^−^, PR^−^) and MDA-MB-231 human adenocarcinoma (HER2−, ER−, PR−) were purchased from American type culture collection (ATCC). PyMT-B01 metastatic murine cells (ER^+^, luminal B) were established from a transgenic MMTV-PyMT B6 tumor.^79^ Dulbecco’s Modified Eagle Medium (DMEM, Gibco) containing 10% FBS and Penicillin (100 units/ ml) and streptomycin (100 μg/ ml) was used for cell culture and seeding. Cell studies were performed with transparent black bottom or white opaque 96 well plates for cell viability or death assays. MatTek dish was used for uptake studies. ^18^FDG was produced by the Washington University in St Louis Cyclotron Facility and used in a radioactive material (RAM) approved room. Cells were seeded with 100 μl DMEM media with and without Phenol red in fluorescence-based cell viability and mechanism of cell death assay, at 6000–8000 cell density per well and cultured for 22 hours in a 37 °C incubator with 5% CO_2_ before each test.

### Receptor target gene and cell surface protein expression

Real-time quantitative polymerase chain reaction (RT-qPCR) was used to evaluate the mRNA gene expression of the target receptor. Transferrin receptor (CD71) and glucose transporter 1 (GLUT-1) were assayed after culturing about 8000 cells for 24 h. Total RNA from the cells was isolated with the RNeasy Mini Plus Kit (Qiagen, Valencia, CA). Complementary DNA was prepared using the SuperScript II First-Strand synthesis system for RT-PCR (Invitrogen, Carlsbad, CA). qPCR was performed using the SYBR Advantage Mix (Bio-Rad) with human and mouse-specific primers, as listed here. Human primers: Gapdh, forward 5’- GGT GTG AAC CAT GAG AAG TAT GA-3’, reverse 5’- GAG TCC TTC CAC GAT ACC AAA G-3’. Tfrc (CD71), forward 5’- TTT CCA CCA TCT CGG TCA TC-3’, reverse 5’- GGG ACA GTC TCC TTC CAT ATT C-3’. Slc2a1 (GLUT-1), forward 5’- GGA CAG GCT CAA AGA GGT TAT G-3’, reverse 5’- AGG AGG TGG GTG GAG TTA AT-3’. Mouse primer: Gapdh, forward 5’- TTCACCACCATGGAGAAGGC-3’, reverse 5’- GGCATGGACTGTGGTCATGA-3’. Tfrc (CD71), forward 5’- AGA CTC TGC TTT GCA GCT ATT-3’, reverse 5’- CCA GTT TCA CAC ACT CCT CTT-3’. Slc2a1 (GLUT-1), forward 5’- TAT CAG CCA CTC TCC TAT CTC C-3’, reverse 5’- AGG TCC AGC CCT ACA GAT TA-3’. All three breast cancer cells were checked for cell surface receptor target expression using a flow cytometry assay performed after 48 h of cell culture in a T-75 flask. Cells at 80% confluence were detached with trypsin EDTA (0.05%) solution after a 1x wash with PBS and then resuspended with DMEM media for centrifugation at 23 °C, 1000 rpm for 3 minutes. After removing the supernatant, the cells were resuspended in 700 μl of UltraCruz Blocking Reagent (Santa Cruz Biotechnology, Inc., Dallas, TX, USA) and incubated in ice for 7 minutes. Each cell line was subsequently counted and aliquoted into six 5 mL Falcon round bottom test tubes, with snap cap, sterile (#352063, Corning Inc., Austin, TX, USA) at 500,000 cells per tube were seeded for the following groups: untreated, untreated plus 4’,6-Diamidino-2-Phenylindole, Dihydrochloride (DAPI, 5 μΜ, #D1306, Invitrogen Corp. Austin, TX, USA). FITC anti-human CD71 Antibody (#334103, BioLegend, San Diago, CA, USA), FITC Mouse IgG2a, κ Isotype Ctrl Antibody (#400207, BioLegend, San Diago, CA, USA) for MDA MB231; APC anti-mouse CD71 Antibody (#113819, BioLegend, San Diago, CA, USA) and APC Rat IgG2a, κ Isotype Ctrl Antibody (#400511, BioLegend, San Diago, CA, USA) for 4T1; and PyMT-BO1 using a 1:700 dilution in a 1:1 Ultra Cruz blocking reagent to 1X PBS solution. Another set of three tubes was seeded for the untreated cells plus DAPI, untreated-DAPI plus secondary antibody only and the treated group with primary and secondary Abs plus DAPI. The primary recombinant anti-glucose transporter GLUT1 antibody (1:250, #ab115730, Abcam, Cambridge, MA, USA) was incubated with cells for 20 minutes in ice, followed by centrifugation at 1200 rpm for 4 minutes. Next, cells were incubated with the secondary Ab, Goat anti-rabbit IgG (H+L) Dylight 550 conjugate goat anti-rabbit IgG (H+L) secondary antibody (1: 500, # 84541, Invitrogen Corp. Austin, TX, USA) for 30 minutes in ice. At the end of the conjugated-Abs or secondary antibody incubation in ice, cells were spun down using the above centrifugation parameters, re-suspended in cell staining buffer (#420201, BioLegend, San Diago, CA, USA) containing DAP1 for 15–20 minutes before a rinse and re-suspension of cells with the cell staining buffer for flow cytometry analysis (BD Biosciences LSRFortessa^™^ Cell Analyzer, Franklin Lakes, NJ, USA).

### In vitro TiO_2_-TC-Tf cellular uptake and subcellular biodistribution

Three breast cancer cell lines (4T1, MDA-MB231 and PyMT-BO1) were seeded at 4000 cells per well of an 8-well microscopy plate Falcon^™^ Chambered Cell Culture Slides (#08-774-208, Fisher Scientific Co. L.L.C., Pittsburgh, PA, USA) with DMEM media supplemented with 10% Fetal Bovine Serum (FBS), penicillin (100 units/ ml), and streptomycin (100 μg/ ml). Cells were cultured overnight at 37°C in a 5% CO_2_ incubator. Fresh Titanium dioxide and titanocene (MilliporeSigma, St Louis, MO, USA), human Apo-transferrin (Athens Research and Tech, Inc., Athens, GA, USA), and Alexa Fluor 680 human transferrin (Invitrogen Corp. Austin, TX, USA) were used to synthesize a fresh batch of the TiO_2_-TC-Tf and Alexa Fluor 680-Tf-TiO_2_ (AF680-Tf-TiO_2_) for each experiment using a previously established protocol.^12^ For the cellular uptake study, old media were removed and replaced with 200 μl of culture media containing control Alexa Fluor 680 transferrin only (AF680-Tf, same concentration used for the AF680-Tf-TiO_2_ conjugated) and AF680-Tf-TiO_2_ conjugate (125 μg/ ml), into each cell type. Cells were incubated at 37 °C in a 5% CO_2_ incubator for 24 h for adequate internalization, followed by media removal, 2x rinse with 1X PBS, and a 30-minute counter staining of cells with SYTO-13 nuclear stain (ThermoFisher Inc, Dallas, TX, USA). After SYTO-13 containing media removal and a 1x rinse with PBS, cells were resuspended in DMEM culture media for live-cell confocal microscopy (FV1000, Olympus Corp, Shinjuku-ku, Tokyo, Japan) using 60x water immersion objective lens with Ex/Em laser of 644/710 nm and 488 / 550 nm wavelength for AF680-Tf-TiO_2_and SYTO 13 nuclear stain, respectively. Images were processed with image J software (U. S. National Institutes of Health, Bethesda, Maryland, USA).

### In vitro RaST cytotoxicity

We developed for the first time the in-vitro RaST dye exclusion cytotoxicity assay in a T25 flask using the following protocol: The three breast cancer cell lines, MDA-MB231, PyMT-BO1, and 4T1, were seeded at 850,000 cells per T25 flask for the cytotoxicity conditions above using a 4 ml solution of DMEM complete growth media. After an overnight culture of cells, 2 ml of the media was removed from the TiO_2_-TC-Tf only and RaST group flask and replaced with a 2 ml complete media containing 0.25 mg/ml of the TiO_2_-TC-Tf into both flasks for overnight incubation at 37 °C, 5% CO_2_ incubator. After 24 h, ^18^FDG (111.0 MBq/ 3.0 mCi) was added to the ^18^FDG only and the RaST group using 300 μl volume. The RAM-containing flask was then moved to a RAM-approved incubator for the final 48 h incubation. The culture media was removed, cells were washed once with 1X PBS, rinsed with 1.5 ml of 0.05% trypsin-EDTA, and then incubated at 37 °C, 5% CO_2_ incubator for 2–3 minutes for cells to detach. Each flask was then supplemented with 2 ml of complete culture media and followed by a cell count using a 1:1 combination of 10 μl of the suspended treated cells and 0.4% trypan blue solution mixed in a 1.5 ml microcentrifuge tube. Finally, 10 μl of the cell:trypan blue solution mix was aspirated and used to fill two or three wells of the Countess cell count instrument (Life Technology Corp. Carlsbad, CA, USA) and read using the standardized set parameters for live and dead cells. Other parameters, like the dead and live-cell size, were captured. Prism software was used to analyze the data for the three consecutive experiments (n=2 per cell line).

### In vitro RaST mechanism of death

RaST cell death mechanism was assayed using a RealTime-Glo^™^ Annexin V Apoptosis and Necrosis Assay (Promega Corp., Madison, WI, USA) according to the manufacturer protocol but with some modifications, and the Western blot assay for necroptosis signaling proteins. Two black clear bottom 96 well plates for the ^18^FDG and non-^18^FDG plates were seeded with 8000 PyMT-B01 and 4T1 cells per well with DMEM culture media supplemented with 10% FBS and P/S in triplicate for the following groups: untreated (control), TiO_2_-TC-Tf only, ^18^FDG only, and RaST and cultured at 37 °C in a 5% CO_2_ incubator. After a 24 h cell culture, 90% of the culture media was removed from the wells and then replaced with 100 μl of fresh phenol red-free DMEM complete media containing 2x mixture of apoptosis and necrosis assay. After 2 h incubation at 37 °C, a pre-treatment relative luminescence unit (RLU) and mean fluorescence intensity (MFI) at 482/20 nm excitation and 528/20 nm emission wavelength were read with the plate reader (Agilent BioTek Synergy Neo2, Santa Clara, CA, USA) simultaneously. Later, the TiO_2_-TC-Tf only and TiO_2_-TC-Tf plus ^18^FDG (RaST) wells received TiO_2_-TC-Tf complex (200 μg/ml final concentration containing 30 μg of total titanium from the working stock concentration of 1 mg/ml), followed by 2 h (4 h post-assay addition) and 21 h post- TiO_2_-TC-Tf (23 h post-assay) addition RLU/MFI read-out. Finally, the ^18^FDG only and RaST wells received a 20 μl of 3.7 MBq (100 μCi) ^18^FDG at 24 h post-TiO_2_-TC-Tf (26 h post-assay), while untreated control and TiO_2_-TC-Tf only wells received 20 μl of PBS vehicle like the ^18^FDG 1X PBS buffered solution vehicle. Other time points include 48 and 52 h post-TiO_2_-TC-Tf addition (50 and 54 h post-assay). For an explanation of the mechanism of cell death, the post-TiO_2_-TC-Tf addition suffix will be used henceforth. The RLU and the MFI values at each time point were summarized with the GraphPad Prism 8-line plot.

Western blot assay samples for the necroptosis signaling proteins used cells treated according to the dye exclusion assay protocol described above with the 4T1 and PyMT-BO1 cells. After 48 h post-^18^FDG treatment, the adherent cells were washed with 1X PBS twice and resuspended in 500 μl homogenization cocktail of 1X RIPA buffer (10mM Tris-HCl, pH 8.0, 140mM NaCl, 1mM EDTA, 0.5mM EGTA, 1% Triton X-100, 0.1% sodium deoxycholate, 0.1% SDS, 1mM PMSF), protease inhibitor and phosphatase inhibitor, scraped, and aspirated into a 1.5 ml micro-centrifuge tube for each of four conditions: untreated, TiO_2_-TC-Tf only, ^18^FDG only and RaST. Cell lysates were homogenized using an ultrasonic processor to sonicate intermittently for less than a minute and incubated in ice for 1 hour. Later, the cell lysate samples were centrifuged at 400 g for 10 minutes at 4 °C. The cell lysates were denatured in SDS gel-loading buffer (100 mM Tris-HCl, 200 mM DTT, 4% SDS, 0.2% bromophenol blue, and 20% glycerol) at 97 °C for 7 minutes and then separated on 12% SDS-polyacrylamide gels (30 to 50 μg of the cell’s lysate proteins per sample). After electrophoresis, all proteins were transferred to PVDF membrane using an EC140 Mini Blot Module (Thermo EC, Holbrook, NY) apparatus. The membrane was blocked for 1 h at room temperature in PBS containing 5% nonfat dry milk and 0.1% Tween-20 (PBS-T), followed by incubation with the primary antibody in PBS-T containing 3% nonfat dry milk at 4°C overnight. After washing three times for 10 minutes each in PBS-T, the membrane was incubated for 1 h with diluted polyclonal horseradish peroxidase (HRP) goat anti-rabbit IgG Ab in PBS-T containing 3% nonfat dry milk (w/v). The membrane was washed three times for 10 minutes each in PBS-T buffer and developed using the chemiluminescence Pierce^™^ ECL Plus Western Blotting Substrate (#32132, ThermoFisher Scientific, Waltham, MA, USA) according to the manufacturer’s instructions. Cell lysates were measured with Bradford protein assay (#5000201, BIO RAD, Hercules, CA, USA), and 30 or 50 μg of each was used for gel loading. Primary antibodies used are: Anti-RIPK1 (1:1000, #A7414, ABclonal, Inc, Woburn, MA, USA), RIPK3 (1: 1000, #A5431, ABclonal, Inc, Woburn, MA, USA), MLKL (1: 1000, #A7414, ABclonal, Inc, Woburn, MA, USA), and NFkB (1:1000, #A5567, ABclonal, Inc, Woburn, MA, USA). The secondary antibody was goat anti-rabbit pAb-HRP (1:3,000, ab205718, Abcam, Cambridge, MA, USA). The estimated target protein bands based on the manufacturer’s recommendation were selected for the qualitative and quantitative analysis with image J software and compared the mean ± SD of each target protein for each treatment group: untreated, TiO_2_-TC-Tf only, ^18^FDG only, and RaST group.

### Animal models

Seven-week-old C57BL/6 mice purchased from Charles River Laboratory (Wilmington, MA, USA) and NSG^™^ or NOD *SCID* IL2 gamma (#005557 Jackson Laboratory, Farmington, CT, USA) were used for either in-vivo biodistribution or therapy studies. All animal experiments complied with the Washington University Animal Welfare Committee’s requirements for the care and use of laboratory animals in research. A 1.0 × 10^5^ cells (Group A) and 5.0 × 10^4^ (Group B) C57BL/6 PyMT-BO1 GFP Luc immunocompetent, and 1.0 × 10^5^ cells NSG PyMT-BO1 GFP Luc immunocompromised orthotopic lower right inguinal mammary fat pad (MFP) model were generated using 40 μl solution of 1:1 PBS (1X) and Matrigel matrix with growth factor (Corning Inc., Austin, TX, USA) containing PyMT-BO1 GFP Luc.^79^

### Inductively coupled plasma mass spectrometry of titanium content

ICP-MS analysis was performed on Perkin Elmer, ELAN DRC II Inductively Coupled Plasma-Mass Spectrometer (ICP-MS) at the Nanotechnology Center, Washington University in St Louis, MO following standard ICP analysis protocol.^17^ All TiO_2_-TC-Tf NPs were freshly prepared for each experiment. Each mouse weighing about 20 g was injected with 100 μg of TiO_2_-TC-Tf NPs in 100 μl of PBS, followed by euthanasia at 24, 48, and 72 h (n=3 per time point) and whole organ excision. Three untreated mice were used for comparative analysis of data. The excised organs such as the spleen, lymph node, lower limbs (femur, tibia, and fibula), lung, and liver were placed in a 5 ml Falcon tube (#352063, Corning Inc., Austin, TX, USA), weighed and stored at −20°C until the time of use. Each pre-weighed sample was transferred into a Teflon reaction vessel containing concentrated nitric acid (1.9 ml) for digestion with the Mars 6 Microwave Digestion System (CEM Corporation). Microwave power was ramped up to 200 °C for 20 minutes, held for 20 minutes at this temperature, and then cooled and diluted to 5 ml 18 MΩ deionized water before the ICP-MS analysis. Additional dilutions are needed for samples above 0.5mg/L. Freshly prepared titanium (Ti) calibration standards of 0.01 μg/l, 0.1 μg/l, 1 μg/l, 10 μg/l, 50 μg/l, 100 μg/l and 500 μg/l were used. 20 ug/L Sc internal standard is added using a sample T. The ICP-MS results were within 10% of the expected concentrations, with a relative standard error of less than 5% for all standards and samples. Ti concentration for each sample was calculated by multiplying the reported Ti concentration by the dilution factor and the final digestion volume of the sample, then dividing it by the tissue weight. The mean ± SD of the Ti concentration for the samples were used for the bar graph plot presentation.

### Bioluminescence imaging

In vivo BLI was performed with an IVIS Lumina (PerkinElmer, Shelton, CT, USA); Living Image 4.3, 5 min to 1 s exposure, bin 2–8, FOV12.5 cm, f/stop1, open filter). Mice were injected intraperitoneally with D-luciferin (150mg/kg in PBS; Gold Biotechnology, St. Louis, MO) and imaged 10 minutes later under isoflurane anesthesia (2% vaporized in O2). Total photon flux (photons/sec) was measured from software-defined contour regions of interest (ROIs) over the MFP tumor using Living Image 2.6.

### Fluorescence molecular tomography

C57Bl/6 orthotopic PyMT B01 GFP Luc mice (nine weeks old, female) with a tumor volume of ~400 mm^3^ were injected with 3.2 mg ml−1 (16 mg kg−1) of TiO2-Alexa680Tf (*n* = 3) synthesized with Alexa680Tf (Life Technologies Inc.) in 1X Dulbecco’s phosphate-buffered saline (DPBS, 100 μl) i.v. through the lateral tail vein. Fluorescence imaging with the FMT imager (Perkin Elmer FMT4000, Waltham, MA, USA) using the excitation and emission wavelengths of 685 nm and 720 nm for whole-animal imaging at pre-injection, 2 h, 6 h, 24 h, 48 h, and 72 h were captured. Mice euthanasia occurred at 24 h (n=1), 48 h (n=1), and 72 h (n=1). Image processing of the tumor site region of interest (ROI) was quantified and analyzed using the FMT analysis package.

### Positron emission tomography and computed tomography (PET/CT).

Small animal PET/CT imaging was done with the Inveon MicroPET/CT scanner (Siemens Medical Solutions, Tarrytown, NY, USA). Three orthotopic C57BL/6 (9 weeks old female) PyMT B01 breast tumor-bearing mice at ~400 mm^3^ tumor size fasted with water for 16 h on days 1, 4, and 7 were tail vein injection with 200 μCi (7.4 MBq)/0.1 ml, 400 μCi (14.8 MBq)/0.1 ml, and 800 μCi (29.6 MBq)/0.1 ml of ^18^FDG, respectively. The PET/CT imager captures static images for 20 minutes, followed by reconstruction with the maximum a posteriori (MAP) method, ^80^ before image co-registration with Inveon Research Workstation (IRW) image display software (Siemens Medical Solutions, Knoxville, TN, USA). Tumor site ROI was selected from PET images using CT anatomical guidelines, and the activity was estimated with the IRW software. The standard uptake values (SUVs) in tumors for each concentration were quantified using the standard SUV formula= [tissue activity (mCiml-1] × [animal weight (g)/ injected dose (mCi).

### RaST-induced necroptosis challenge testing

After treating the PyMT-BO1 GFP Luc cells using the *in vitro dye exclusion* RaST cytotoxicity protocol, cells were trypsinized (0.05% Trypsin-EDTA) and re-suspended in a complete DMEM culture media. A 10 μl cell suspension to 10 μl trypan blue stain (0.4%, #T10282, Invitrogen, Carlsbad, CA, USA), and counted with the Countess instrument (ThermoFisher Scientific, Waltham, MA USA). About 50,000 live PyMT-BO1 GFP Luc cell suspension per mouse in a 1.5 ml microcentrifuge tube for centrifugation at 1500 rpm for 3 minutes at 23°. Cells pellets were then re-suspended in a 1:1 Matrigel and 1X PBS solution for orthotopic MFP implantation, as described above, for the following groups: Untreated, TiO_2_-TC-Tf only, ^18^FDG only, and RaST group. Tumor growth was monitored with caliper and BLI, while the results were analyzed with GraphPad Prism8 software.

### In vivo RaST response

Immune-activating drug responses are best determined using pre-established criteria associated with overall survival information due to individual differential antitumor responses. Here, we adopted the immune-related response criteria (irRC) to determine treatment response based on each mouse antitumor response type (complete response (CR), a partial response (PR), and progressive disease (PD) or non-responding (NR) and the overall survival patterns instead of the response evaluation criteria for solid tumors (RECIST criteria).^45^ A pre-assessment of the tumor growth rate (mm^3^) between days 7 to 22 in both C57BL/6 and NSG PyMT-BO1 GFP Luc model using caliper measurement and tumor weight (mg) assessment on day 19 and 22 post-implantation for NSG and C57BL/6, respectively, were determined and used for in-vivo RaST treatment planning and response prediction. (Supplementary Fig. 4 A-B). For the in vivo RaST experiment, orthotopic MFP female C57BL/6 PyMT-BO1 GFP Luc (Group A- 1.0 × 10^5^ cells, Untreated n=6, TiO_2_-TC-Tf n=7, ^18^FDG only n=7 and RaST n=8, and Group B- 5.0 × 10^4^, untreated n=6, TiO_2_-TC-Tf n=7, ^18^FDG only n=7 and RaST n=7, and NSG PyMT-BO1 GFP Luc (Group A- 1.0 × 10^5^, Untreated n=3, TiO_2_-TC-Tf n=5, ^18^FDG only n=6 and RaST n=6,) mice on day 5 post-implantation were randomly stratified equally into the four experimental groups: untreated control, ^18^FDG only, TiO_2_-TC-Tf complex only, and RaST groups based on their BLI values. The TiO_2_-TC-Tf and RaST group received tail vein injection of 100 μl TiO_2_-TC-Tf (5 mg/ kg, 1 mg/ ml final product) followed by a post-TiO_2_-TC-Tf 24 and 72 h ^18^FDG 22.2 MBq (600 μCi)/ 0.1 ml intraperitoneal (i.p). injection after 6 h fasting. To avoid difficulties in accessing the tail vein for the multiple TiO_2_-TC-Tf injections, we chose the i.p. injection for ^18^FDG. Tumor burden was monitored using a twice weekly caliper for tumor volume estimation using the volume equation (mm^3^) = (Length × Width^2^)/2, and BLI measurements to determine tumor cells’ photon flux. The BLI measurement for tumor response estimation stopped at day 22 due to the bioluminescence signal-dampening effect in the untreated, which may result from intrinsic necrosis, hypoxia, and poor luciferin perfusion due to the tumor growth mass effect. The mice were euthanized when the tumor size reached 2 cm in diameter in any direction by cervical dislocation after anesthesia with 5% isoflurane. The objective treatment response classification of each mouse into the NR, PR, and CR was determined based on these criteria. We defined the PR as tumor volume ≤ 250 mm^3^ (Group B) and ≤ 500 mm^3^ (Group A) on day 22. CR represents a non-palpable and non-BLI detectable tumor,^81^, while NR or progressive disease represents continuous tumor growth throughout the measurement period. The tumor response profile was summarized using individual or Spaghetti graph plot (Supplementary Fig. 5 A-F), part of a whole plot or donut chart, and a Kaplan-Meier Survival plot for survival comparison using GraphPad 8 Prism software.

### Histologic immune cell profiling

To evaluate tumor-immune cell interaction post-therapy, tumor masses were harvested on days 15 and 19 post-implantation from the untreated and RaST group (n=4). The lymph nodes excised during the surveillance period in CR mice. Hematoxylin and Eosin (H&E) staining was performed on the tissues using a standard protocol. Immunofluorescence (IF) performed with Alexa Fluor 647 anti-mouse CD45 Ab (#103123, 1:120, BioLegend, San Diago, CA, USA), Alexa Fluor^®^ 647 Rat IgG2b, κ Isotype Ctrl Ab (#400626, 1:120, BioLegend, San Diago, CA, USA), APC anti-mouse CD8b Antibody (#126613, 1:120, BioLegend, San Diago, CA, USA), APC Rat IgG2b, κ Isotype Ctrl Antibody (#400611, 1:120, BioLegend, San Diago, CA, USA), Alexa Fluor^®^ 647 anti-mouse/human CD11b Antibody (#101220, 1:120, BioLegend, San Diago, CA, USA). The anti-mouse Integrin, alpha X (ITGAX), also known as CD11c Polyclonal Ab (#A1508, 1:500, ABclonal, Tech., Woburn, MA USA) was counterstain with Alexa Fluor 594 donkey anti-mouse (# **R37119, 1: 1000,** ThermoFisher Inc, Dallas, TX, USA) for 22 h incubation at 4°C after a 90-minute blocking with Ultra Cruz blocking solution (Santa Cruz Biotec, Inc, USA). H&E-stained imaging was done with the epi-fluorescence microscope 100x (Olympus BX51, FL, USA). In addition, the IF slide imaging was with the Zeiss Axio scan Z1 slide automated scanner, 20x objective lens (Jena, Germany). Image processing was performed with the free Zeiss software, followed by quantitative analysis of the positively stained cell regions (n=5 for untreated and RaST) at the periphery and core using the image-Pro Premier Software E 10. A pathologist analyzed all histology samples.

### Luminex immunoassay for immune modulation profiling

Mouse whole blood samples obtained from the superficial temporal vein on day 0 (or naïve non-tumor bearing), 12 and day 19 post-tumor implantation and pre-euthanasia from the Group B cohort were immunoassayed using Luminex xMAP technology with a ProcartaPlex Mouse Cytokine & Chemokine Convenience Panel 1 26plex and two additional cytokines (# EPXR260-26088-901, Invitrogen Corp. Austin, TX, USA). Similarly, plasma samples collected on day 19, at pre-euthanasia (from Group A cohort, n= 3–7), and during the surveillance period (Group B cohort, n= 1–2) were assayed for soluble immune checkpoint protein receptors or ligands (ICPRL) using the MILLIPLEX^®^ Human Immuno-Oncology (Human I-O kit) Checkpoint Protein Panel 2 - Immuno-Oncology Multiplex Assay (# HCKP2–11K, MilliporeSigma, Burlington, MA, USA) after a preliminary quality control confirmed eight positive mouse analytes including the four used in this study: LAG-3, TLR-2, PD-L1 and PD-1. Generally, whole blood samples collected in Microvette^®^ 100 EDTA K3E, 100 μl, cap violet, flat base tube (#20.1278.100, SARSTEDT, Inc, Newton, NC, USA) were centrifuged for 10 minutes at 4 °C and 2000 rpm speed within the first 20 minutes after collection. The plasma component was aspirated into a 1.5 ml microcentrifuge tube and stored at −20 °C before processing. These samples preparation, including the quality control samples: recombinant mouse purified protein (#50502-M08B, #50124-M08H, #50010-M03H, #53069-M08H- Sinobiological, Wayne, PA, USA, and #752602, BioLegend, San Diago, CA, USA) for the Luminex assay used a 20 μl from plasma to distilled water (1:2) mixture to seed into the working black 96 microplate and analyzed with the Luminex FLEXMAP3D instrument at Bursky Center for Human Immunology & Immunotherapy core facility at Washington University in St. Louis, MO. ELISA assay results were expressed in picogram/milliliter (pg/ml) for each specimen, and the data were summarized using a subtraction method between the untreated tumor-bearing mouse values and the treatment groups (TiO2-TC-Tf only, ^18^FDG only, and RaST). Naïve non-tumor bearing mouse value was assayed as an additional control.

### Residual tumor identification

All three CR mice underwent whole-body BLI imaging for residual PyMT-BO1 GFP Luc cells on days 57, 140, and 198 post-implantation or surveillance endpoint. To determine the presence or absence of MRT, LS301 (60 μM, 100 μl) formulated in 1% human serum albumin PBS was injected i.v. The ventral, dorsal, and right lateral fluorescence images were acquired using the Pearl NIR fluorescence imager (LI-COR Biotechnology, Lincoln, NE, USA) with the 800 nm channel (Excitation 785 nm and Emission 820 nm) under 1.5 % Isoflurane- oxygen gas anesthesia with a flow rate of 0.8– 1.0 liter/min followed by cervical dislocation under Isoflurane anesthesia. After euthanasia, a mid-line skin incision followed by skin retraction to expose the underlying tissues for in situ imaging. LS301 fluorescent LNs in the ipsilateral and contralateral lower inguinal MFP were collected and histologically analyzed using fluorescence, H&E, and IF antibody staining in the tissues was performed with Rat anti-Polyoma virus, Medium T (PyMT) antigen Ab (1:250, #ab15085, Abcam, Cambridge, MA, USA) and Cy5 Goat anti-rat IgG secondary Ab (1:500, # **A10525** Invitrogen, Carlsbad, CAUSA). Also, Alexa Fluor 647 anti-mouse CD45 Ab (#103123, 1:120, BioLegend, San Diago, CA, USA) and Alexa Fluor^®^ 647 Rat IgG2b, κ Isotype Ctrl Ab (#400626, 1:120, BioLegend, San Diago, CA, USA). were used for the IF staining and imaged with an epi-fluorescence microscope (Olympus BX51, FL, USA).

### Statistics analysis

Results were summarized using a mean and standard error of the mean (mean ± SEM or SD) and statistical testing using either a student t-test with or without Mann-Whitney post-test or one-way ANOVA (non-parametric) and followed by Kruskal-Walli’s test and Dunn’s multiple comparison post-test. Statistical significance was determined with a p-value <0.05 *. <0.01 **, <0.001 *** and <0.001 ****.
